# Parallel Potentiometric and Capacitive Response in a Water-Gate Thin Film Transistor Biosensor at High Ionic Strength

**DOI:** 10.3390/s21165618

**Published:** 2021-08-20

**Authors:** Hadi AlQahtani, Abdullah Alswieleh, Ibrahim Al-Khurayyif, Saad AlGarni, Martin Grell

**Affiliations:** 1 Department of Physics and Astronomy, College of Science, King Saud University, Riyadh 11451, Saudi Arabia; IbrahimAl-Khurayyif@outlook.com (I.A.-K.); saaalgarni@KSU.EDU.SA (S.A.); martin@spinne.plus.com (M.G.); 2 Department of Chemistry, College of Science, King Saud University, Riyadh 11451, Saudi Arabia; aswieleh@ksu.edu.sa

**Keywords:** biosensor, WGTFT, SARS-CoV-2, RBD spike protein

## Abstract

We show that an SnO_2_-based water-gate thin film transistor (WGTFT) biosensor responds to a waterborne analyte, the spike protein of the SARS-CoV-2 virus, by a parallel potentiometric and capacitive mechanism. We draw our conclusion from an analysis of transistor output characteristics, which avoids the known ambiguities of the common analysis based on transfer characteristics. Our findings contrast with reports on organic WGTFT biosensors claiming a purely capacitive response due to screening effects in high ionic strength electrolytes, but are consistent with prior work that clearly shows a potentiometric response even in strong electrolytes. We provide a detailed critique of prior WGTFT analysis and screening reasoning. Empirically, both potentiometric and capacitive responses can be modelled quantitatively by a Langmuir‒Freundlich (LF) law, which is mathematically equivalent to the Hill equation that is frequently used for biosensor response characteristics. However, potentiometric and capacitive model parameters disagree. Instead, the potentiometric response follows the Nikolsky-Eisenman law, treating the analyte ‘RBD spike protein’ as an ion carrying two elementary charges. These insights are uniquely possible thanks to the parallel presence of two response mechanisms, as well as their reliable delineation, as presented here.

## 1. Introduction

Following the invention of the ‘ion-sensitive field effect transistor’ (ISFET) by Bergveld in 1970 [1], the sensor community has developed an entire ‘family’ of field effect-based potentiometric sensors for waterborne (aqueous) analytes. This now includes field effect sensors for biomedical analytes far beyond the initial cations, with wide applications as ‘BioFETs’, reviewed e.g., in [2,3]. In the ISFET concept, a sensitive element (‘sensitiser’, ‘receptor’, or ‘recognition element’) is applied to the gate contact or the semiconducting film of a thin film field effect transistor (TFT) that acts as a transducer when operated and characterised while in contact with an aqueous medium. If the medium carries the analyte for which the sensitiser selects, selective binding of the analyte to the sensitiser leads to an interface potential that shifts the TFT threshold voltage (V_T_). This allows quantitative evaluation of the analyte concentration via electrical characterization of the TFT. A shift in V_T_ leads to a change in the TFT’s drain current I_D_ under constant gate (V_G_) and drain (V_D_) voltages. Hence, e.g., a normalised change in I_D_ can serve as a generic amperometric sensor metric. A more detailed characterisation reveals the underlying potentiometric threshold shift ΔV_T_ versus analyte concentration (c) that can be modelled against theoretical response laws.

A major advance on the original ISFET transducer was the development of the ‘extended-gate field effect transistor’ (EGFET, [4]). The EGFET separates the sensitive element into an external electric double layer (‘EDL’) capacitor cell filled with the aqueous solution under test. The aqueous solution acts as an electrolyte that communicates potentials over macroscopic distances (millimetres or more) with high capacitance via the formation of a pair of electric double layers (EDLs) at opposing metal/electrolyte interfaces. The gate of an unmodified stock TFT is then addressed across the EDL capacitor. However, the EGFET is limited to a potentiometric response, i.e., the cause of any sensor response to analyte is a c-dependent interface or membrane potential. This is true even if ΔV_T_ is read indirectly via I_D_.

The ISFET discipline was re-invigorated by a report in 2010 of an organic ‘water-gate thin film transistor‘ (WGTFT) by Horowitz et al. [5]. The WGTFT incorporates an electric double layer (EDL) capacitor into the transistor itself, and uses the aqueous sample as an active part of the field effect device. This report led to intense research into the WGTFT as a new transducer concept within the ISFET sensor family. Within the WGTFT platform, researchers introduced a range of sensitisers for the detection of waterborne analytes, including ions, as well as organic pollutants, hormones, and viruses. Sensitisers may be immobilised onto the WGTFT’s gate electrode [6,7,8] or semiconducting channel [9,10], introduced via a plasticised PVC membrane that separates the electrolyte into two compartments [11,12,13], or even dispersed into an organic semiconducting film itself [14]. Sometimes, the semiconductor itself may be sensitive, e.g., to pH [15]. As the WGTFT uses the electrolyte as gate medium, not only an interface potential would affect I_D_ (via V_T_), but also a change in the specific capacitance C_i_ of the gate medium, cf. Equation (1) in Section 4. In principle, this opens a second, capacitive sensing window in addition to the potentiometric mode to which ISFET and EGFET are limited. Characteristics of WGTFT sensors for inorganic ions and small organic molecules are accounted for by a solely potentiometric response, e.g., [6,8,11,12], and often can be fitted well to established potentiometric response laws. However, when the sensitiser is a large protein, e.g., an antibody that sensitises for a virus, a dominantly or almost exclusively capacitive response mode was claimed for WGTFTs using an organic semiconductor, e.g., [7,9,16]. The absence of a potentiometric response was explained by electric screening in strong electrolyte. However, we find this explanation inconsistent, as discussed in detail below in this introduction, and in Section 5.

We present here a WGTFT biosensor with a parallel potentiometric and capacitive response despite using a strong electrolyte as a gate medium, together with an improved quantitative understanding. Instead of the more common organic WGTFT [6,7,9,11,14,16], we use the precursor-route metal oxide SnO_2_ as a WGTFT semiconductor. SnO_2_ is far cheaper than organic semiconductors, avoids aromatic or chlorinated processing solvents, and is known for its good stability and low threshold in the WGTFT sensor platform [8,12,13,17]. SnO_2_ is immune to electrochemical doping that is undesirable in WGTFT sensors, but sometimes occurs with organic semiconductors in strong electrolytes [18], and may show less dependency of mobility on V_G_. Most importantly, we introduce a new analysis of WGTFT response characteristics, based on output rather than transfer characteristics. Prior works, including [7,9,16], use the transfer characteristics, i.e., the gate voltage sweep I_D_ (V_G_) (V_D_ = const.), and its derivative, the transconductance g_m_ = ∂I_D_/∂V_G_, to quantify V_T_ and C_i_, cf. Equation (1) and our discussion in Section 3. However, in particular in organic semiconductors, carrier mobility (μ) is known to depend on gate voltage, e.g., [19], but must be assumed to be constant to delineate V_T_ and C_i_ from transfers. Singh et al. [20], among others, describe how V_G_-dependent μ leads to false evaluation of V_T_ from transfer characteristics. Further, DC transistor characteristics can only reveal the mobility-capacitance product μC_i_ (cf. Equation (1) below), which is taken as a measure of C_i_. This is justified only under the assumption of constant μ, but this again is questionable if μC_i_ is evaluated from a transfer characteristic where gate voltage is swept. Moreover, C_i_ of an EDL itself may depend on gate voltage [21], further undermining analysis. We thus consider our analysis based on the linear regime of output (constant gate voltage) characteristics of an inorganic WGTFT more reliable. By analysing the linear regime only, we avoid pinch-off in the channel that occurs at saturation, and would lead to a strong gate voltage gradient along the transistor channel. As a contemporary (but arbitrary) example, we have sensitised our WGTFT at the gate contact with the antibody (immunoglobuline IgG1) against the SARS-CoV-2 virus. The immunoglobuline selectively binds to the SARS-CoV-2 virus via the viruses’ superficial RBD spike protein, and we here characterise this WGTFT under exposure to the isolated RBD spike protein of the SARS-CoV-2 virus, rather than the SARS-CoV-2 virus itself. This avoids working with a high-level biohazard, while still serving the purpose of our study, which is the detailed investigation of response modes in WGTFT biosensors. Moreover, we work at rather high spike protein concentrations to ensure a strong response. We indeed find a strong impact of spike protein concentration on WGTFT characteristics. To delineate potentiometric (V_T_) and capacitive (C_i_) response, we employ a new analysis based on output rather than transfer characteristics. We establish that potentiometric and capacitive sensing modes operate in parallel, in particular, there is no screening of the potentiometric response. Both responses reduce I_D_, hence a simple amperometric (I_D_- based) sensor metric is amplified by the parallel response. We establish separate smooth calibration charts for amperometric, potentiometric, and capacitive sensor metrics against spike protein concentration, and fit them against established response laws. Finally, we provide a detailed critique of the previously reported apparent absence of potentiometric response in WGTFT biosensors, which we find unsound both theoretically and experimentally, and issue recommendations for future work with transistor-based biosensors.

## 2. Materials and Methods

### 2.1. Preparing WGTFT Substrates

As TFT contacts, we deposited a set of five contact pairs by thermal evaporation (base vacuum ~10^−6^ mbar) using a shadow mask onto 15 mm × 20 mm flat quartz glass substrates sourced from Ossila Ltd. (order code S151). An adhesion layer of Cr (20 nm) was deposited first followed by Au layer (200 nm). Each substrate contains five pairs of electrodes separated by a channel with a length of L = 30 μm and a width of W = 1000 μm (^W^/_L_ = 33.3). For the semiconducting channel, we largely followed the precursor route described, e.g., in [12]; that is, we sprayed a 17.5 g/L solution of SnCl_4_ ⋅5H_2_O onto contact substrates heated to 430 °C using an airbrush from a distance of about 20 cm. This was followed by ten similar sprays (3 to 5 s) with 2 min intervals. Afterwards, the substrates were left for 1 h on the hotplate (at 430 °C) for full decomposition of SnCl_4_ into the electron-transporting semiconductor SnO_2_. The decomposition releases HCl as a by-product, which is highly volatile at 430 °C.

### 2.2. Sensitising the WGTFT Gate Contact

**Figure 1:**
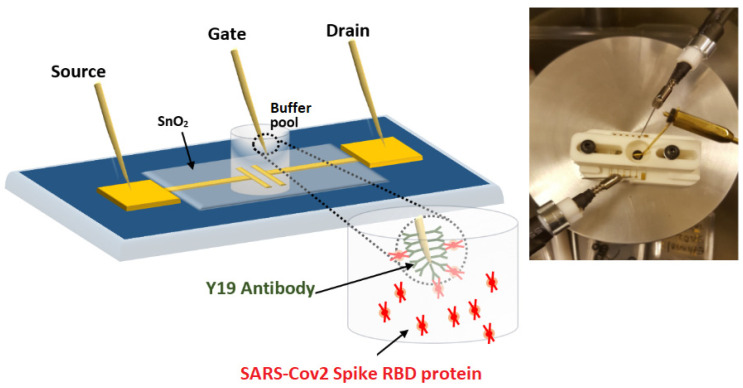
Schematic view of our WGTFT biosensor. ‘Y19′ stands for the antibody (immunoglobuline, IgG1) specific to the SARS-CoV-2 virus. The inset shows the practical implementation in a 3D-printed gating pool.

As gate contacts, we used gold plated needles sourced from [22]. Needles were bent into L-shape and sensitised in two steps. First, we washed needles with water and ethanol, then incubated them in 10 mL of 1 mmol/L cysteamine in ethanol for 24 h at ambient conditions. Cysteamine-functionalized needles were washed thoroughly with ethanol and dried under nitrogen stream, and kept in ethanol for further use. The second step is the immobilisation of an immunoglobuline (antibody) onto the cysteamine-functionalized Au needle. We sourced both the RBD spike protein of the SARS-CoV-2 virus [23] and its specific antibody [24], a recombinant monoclonal human immunoglobuline (IgG1), from InvivoGen. We immobilised IgG1 (‘Y19’ in Figure 1) on the cysteamine-functionalised gate contact by first activating carboxyl groups of IgG1 with N-(3-Dimethylaminopropyl)-N′-ethylcarbodiimide (EDC) (20 mmol/L) and N-Hydroxysulfosuccinimide sodium salt (NHS) (20 mmol/L) in PBS buffer solution for 60 min at room temperature. Then, a cysteamine-functionalised gold-plated WGTFT contact needle was immersed in 2 mL activated IgG1 solution for 3 h to covalently immobilise the antibody. Finally, the sensitised needle was washed with PBS buffer solution and stored in PBS buffer at −20 °C for further use.

### 2.3. Contacting and Characterising the WGTFT Sensor

We fitted SnO_2_ WGTFT substrates in a 3D printed holder with a pool of >500 μL capacity, as shown in the inset in Figure 1. The pool was filled with 500 μL Certipur^®^ phosphate buffer solution sourced from Sigma Aldrich, Cat No 1094391000, pH = 7.0, ionic strength i_s_ = 170 mmol/L, Debye length 0.74 nm. Certipur is closely related to conventional PBS buffer, but biomedical samples were found to be particularly stable in Certipur [25]. We used two micropositioners (sourced from microxact) fitted with tungsten to contact the source and drain ports, and a third micropositioner to immerse the L-shaped end of a sensitised gate needle into the PBS pool. We addressed the WGTFT and recorded both output and transfer characteristics using a Keithley dual source-unit (2634B) with Keithley Kickstart software. For output characteristics, we recorded the WGTFT drain current I_D_ upon sweeping the drain voltage V_D_ from 0 to 1 V in steps of 20 mV at a fixed value of the gate voltage V_G_ = 0.3 V. For transfer characteristics, we recorded I_D_ upon sweeping the gate voltage V_G_ from −0.4 V to 0.5 V in 20 mV steps at fixed V_D_ = 0.2 V. We first recorded characteristics with a gate pool filled with 500 µL PBS buffer only. Then, we added RBD spike protein to match the IgG1 sensitiser, also sourced from Invivogen [23], at a concentration of 100 µg/mL (50 µg dissolved in 0.5 mL of endotoxin-free water). The spike protein simulates the SARS-CoV-2 virus itself at a much-reduced biohazard level. We pipetted aliquots of 2 µL, 4 µL, and 8 µL up to a total of 64 µL from the spike protein solution into the sample pool, giving a concentration range (0.4 to 11.3) µg/mL of the spike RBD protein in the sample pool. This relatively large concentration range (compared, e.g., with [26]) was chosen to ensure a strong response. After each addition step, we waited 5 min, then recorded the WGTFT characteristics. In a control experiment, we added 40 µL of additional PBS buffer to the gate pool to confirm adding more electrolyte itself does not lead to a change in WGTFT characteristics. This control experiment is shown as Supplementary Materials Section S1. Selectivity of the RBD spike/specific antibody interaction was established by the supplier on one example [24]. However, given the large number of potential interferants in vivo, a full selectivity survey would be a near-infinite task. Here, we rely on the assumption that selective ‘lock and key’ recognition between virus RBD spike protein and specific antibody is established by the evolution of the antibody. Moreover, even if an interferant did exist, this would not compromise the conclusions of our work, which concern the WGTFT’s response mechanism.

The overall setup is illustrated in Figure 1.

## 3. Results

### WGTFT Characteristics under Spike Protein Exposure

**Figure 2:**
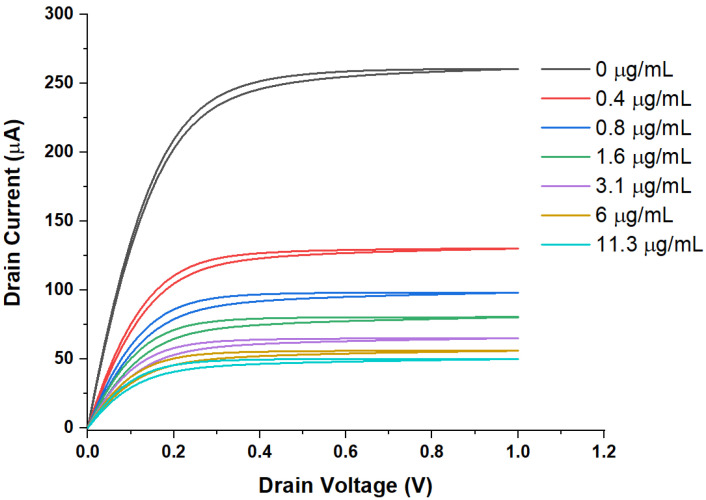
Output characteristics under increasing spike protein concentration, c. Note, all outputs are taken at V_G_ = 0.3 V, and only c varies.

**Figure 3:**
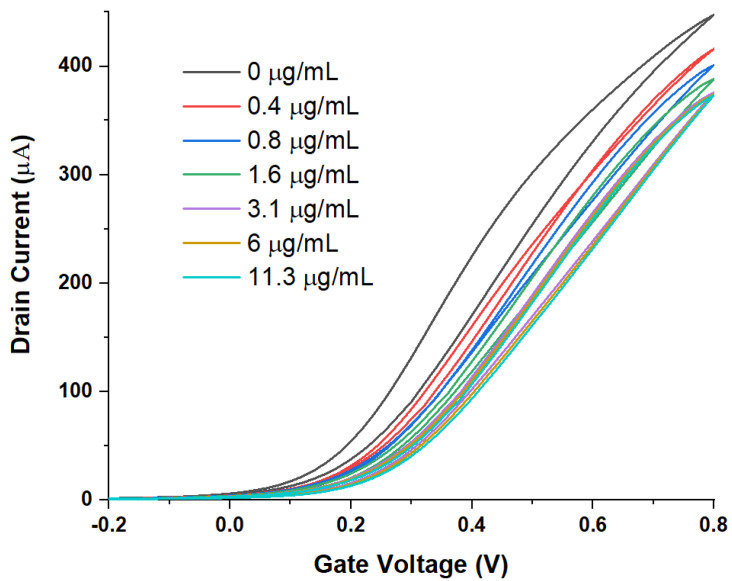
Transfer characteristics under increasing concentration (V_D_ = 0.2 V). The same transfers are shown on a log I_D_ scale in the Supplementary Materials Section S2.

Figure 2 and Figure 3 show the output and transfer characteristics of SnO_2_ WGTFTs when RBD spike protein concentration is increased in several steps by titrating concentrated RBD solution into the gating pool that initially holds PBS without the spike protein.

All characteristics show a drain current for positive V_G_ and V_D_, as expected for a transistor with an electron-transporting semiconducting channel. All output characteristics follow the general shape predicted by the standard TFT equation, Equation (1) below, for TFTs at gate voltages above the threshold: A linear increase in I_D_ with V_D_ at small V_D_, flatlining into a saturated drain current I_D,sat_ at higher V_D_. Clearly, both output and transfer characteristics respond to the addition of spike protein to the sample pool. This suggests that RBD spike protein binds to the antibody immobilised on the gate contact. The transfers visibly display a threshold shift towards more positive gate voltages, i.e., the response mechanism is at least in part potentiometric. A parallel capacitive response mechanism cannot be assessed from first inspection, but requires quantitative analysis. However, overall, the response is more clearly visible in the output characteristics, which also show smaller hysteresis.

## 4. Discussion

### 4.1. Generic Evaluation of WGTFT Response to RBD Spike Protein

**Figure 4:**
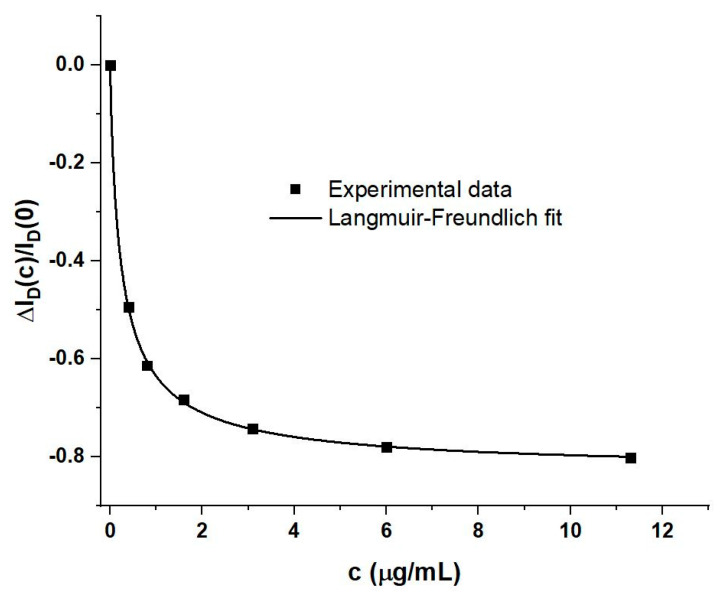
Amperometric response metric ΔI_D_(c)/I_D_(0) vs. spike protein concentration, c. The ‘Langmuir‒Freundlich’ fit line is drawn according to Equation (6) below; fit parameters are included in table in Section 4.3.

A generic quantitative WGTFT response metric is given by the relative change in drain current, I_D_, under increasing spike protein concentration, read at fixed gate and drain voltages, V_G_ and V_D_. The easy-to-read amperometric metric, ΔI_D_(c)/I_D_(0), is commonly used to evaluate WGTFT biosensors, e.g., [7,10]. Note that this does convolute the potentiometric and capacitive response into a single metric. We here read ΔI_D_(c)/I_D_(0) from output characteristics at V_G_ = V_D_ = 0.3 V. Figure 4 shows a plot of this amperometric metric against spike protein concentration, c.

Figure 4 provides a calibration for the WGTFT sensor. We used OriginLab software to fit data to a model based on the Langmuir‒Freundlich (LF) adsorption isotherm, to be discussed in detail in Section 4.3 below. The model is, at this stage, used only because it empirically provides a good fit, and allows some quantitative conclusions. With the help of the analytic Equation (6a) with the model parameters fitted against measured data, as listed in table in Section 4.3, we can determine a limit-of-detection (LoD). An appropriate procedure is described in [17] and is applied to our data in the Supplementary Materials Section S3. As a result, we find an LoD of c_LoD_ = 6.2 ng/mL RBD spike protein.

### 4.2. Delineating Capacitive and Potentiometric WGTFT Response

Above the threshold, |V_G_| > |V_T_|, WGTFT drain current is related to gate and drain voltage by the widely used field effect transistor equations, Equation (1), e.g., [27]:

I_D_ = ^W^/_L_ μC_i_ (V_G_ − V_T_ − V_D_/2) V_D_ for |V_D_| < |V_G_ − V_T_| (1a)

I_D_ = I_D,sat_ = ^W^/_2L_ μC_i_ (V_G_ − V_T_)^2^ for |V_D_| > |V_G_ − V_T_| (1b)

Therein, W/L is the width/length of the semiconducting channel, μ is the charge carrier mobility in the transistor channel, C_i_ is the specific capacitance (capacitance/unit area) of the gate medium, and V_T_ the threshold voltage. The regimes |V_D_| < |V_G_ − V_T_| and |V_D_| > |V_G_ − V_T_| are called ‘linear’ and ‘saturation’ regimes, respectively. Note that, within the linear regime, |V_D_| < |V_G_ − V_T_|, I_D_ is linear with V_G_. I_D_ is linear with V_D_ only in the stricter limit |V_D_| << |V_G_ − V_T_|.

Equation (1) shows that drain current can, in principle, be affected in three ways by the binding of an analyte from the gate electrolyte to a sensitiser immobilised within the WGTFT architecture: by an impact on carrier mobility, on specific capacitance, or on threshold voltage. Note that mobility and specific capacitance appear in the equation only as their product, μC_i_. Careful analysis of measured WGTFT characteristics allows, in principle, to delineate between threshold voltage (potentiometric response) and the mobility‒capacitance product, μC_i_. Delineation between mobility and capacitance is not possible with DC methods alone. However, when the sensitiser is immobilised on the gate contact, it is spatially far removed from the semiconducting channel, and an impact of analyte/sensitiser binding on carrier mobility is thus generally discounted, e.g., [7]. Even in the case of antibody immobilisation on the semiconducting film [9,10], the large size of the antibody spatially separates the analyte/sensitiser binding site from the semiconducting channel. It was thus assumed that mobility is not affected by analyte/sensitiser binding and the mobility‒capacitance product thus may only change as a result of a change in specific capacitance, and can serve to quantify the capacitive response.

Practically, the delineation of WGTFT parameters V_T_ and μC_i_ from measured characteristics is not trivial. Here, we avoid the common quantitative evaluation of transfer characteristics for the reasons given in the introduction. Instead, we introduce an alternative procedure to delineate V_T_ and μC_i_ based on output characteristics. Output characteristics are recorded at constant V_G_, avoiding artifacts from gate-dependent μ and C_i_. We re-plot the output characteristics, Figure 2 (omitting hysteresis, only the rising V_D_ half-cycle is used), in the form of I_D_/V_D_ versus V_D_. We drop the I_D_ = V_D_ = 0 data point to avoid ill-defined division. For WGTFT in the linear regime, |V_D_| < |V_G_ − V_T_|, Equation (1a) predicts a straight line with negative slope in the I_D_/V_D_ versus V_D_ plot, according to Equation (2):

I_D_/V_D_ = ^W^/_L_ μC_i_ (V_G_ − V_T_) − ^W^/_2L_ μC_i_V_D_ (2)

**Figure 5:**
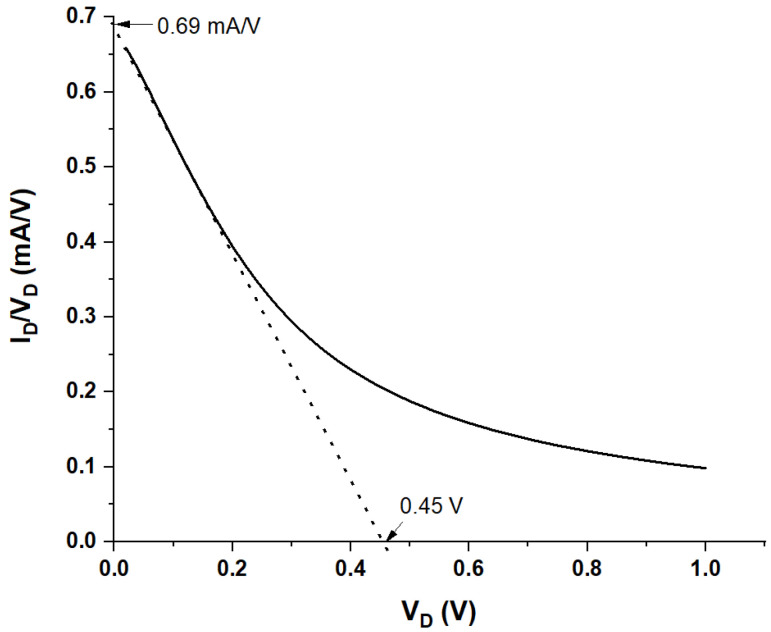
Example of the evaluation of outputs according to Equation (2). c = 0.8 μg/mL. V_DX_ = 450 mV, (I_D_/V_D_)_X_ = 0.69 mA/V.

As saturation is reached, Equation (1b) applies, and the I_D_/V_D_ versus V_D_ plot curves away from the straight line. We fit a straight line to (only) the data points that line up straight and extend this line to its intercepts with the V_D_-axis and the I_D_/V_D_ axis. We read the intercepts as V_DX_ and (I_D_/V_D_)_X_. An example of this type of plot, and line fitting, is shown in Figure 5 below.

At V_DX_, (I_D_/V_D_) → 0, and Equation (2) reads as follows:

0 = −^W^/_2L_ μC_i_V_DX_ + ^W^/_L_ μC_i_ (V_G_ − V_T_) ⇒ V_T_ = V_G_ − V_DX_/2 (3)

Hence, from Equation (3), we find threshold voltage, V_T_, from the intercept V_DX_.

Further, at (I_D_/V_D_)_X_, V_D_ → 0, and Equation (2) reads as follows:

(I_D_/V_D_)_X,_ = ^W^/_L_ μC_i_ (V_G_ − V_T_) ⇒ ^W^/_L_ μC_i_ = (I_D_/V_D_)_X_/(V_G_ − V_T_) (4)

Equation (4) allows the calculation of ^W^/_L_ μC_i_ from (I_D_/V_D_)_X_ and the previously determined threshold voltage, V_T_. ^W^/_L_ is a dimensionless geometry constant (here, ^W^/_L_ = 33.3), and carrier mobility μ in the semiconductor is assumed not to be affected by analyte/sensitiser binding, hence ^W^/_L_ μC_i_ is proportional to the specific capacitance, C_i_, of the WGTFT’s gate medium. The above analysis of measured output characteristics can thus delineate and quantify the potentiometric response, shown as a change in V_T_, and capacitive response, shown as a change in ^W^/_L_ μC_i_. We repeat this analysis for every output characteristic shown in Figure 2 to find V_T_(c) and ^W^/_L_ μC_i_(c). Further, we define concentration-dependent potentiometric, as well as capacitive, response metrics in Equation (5):

ΔV_T_(c) = V_T_(c) − V_T_(c = 0) (5a)

ΔC_i_(c)/C_i_(0) = [C_i_(c)/C_i_(c = 0)] − 1 = [^W^/_L_ μC_i_(c)/^W^/_L_ μC_i_(c = 0)] − 1 (5b)

Both response metrics are defined so that they are equal to zero for c = 0. Equation (5b) corresponds to the transconductance-based response metric Δg_m_/g_m_(0) introduced by Bortolotti et al. [7]. Unlike Bortolotti et al., we do not normalise the ΔV_T_(c) sensor metric to ΔV_T_(c)/V_T_(c = 0). Theoretical models of potentiometric response (e.g., Equation (7) below) quantify ΔV_T_(c) rather than ΔV_T_(c)/V_T_(c = 0). Moreover, V_T_(c = 0) ≈ 0 is possible, and in fact, we find our V_T_(c = 0) is very close to zero. Both theory and experiment thus discourage normalising the potentiometric sensor metric to V_T_(c = 0).

Table 1 below summarises results of our analysis of the output characteristics shown in Figure 2.

The mobility‒capacitance product cannot be separated from the output or transfer measurements alone, and separation is not essential for our conclusions here. However, mobility can be calculated if an independent value for C_i_ can be established. A detailed discussion is presented in Supplementary Materials Section S4, giving an estimate of μ = 59 cm^2^/Vs.

Table 1 clearly shows both a potentiometric and a capacitive response in parallel. In particular, there is no evidence that the potentiometric response is screened, despite the use of a high ionic strength electrolyte, and the large separation between analyte/sensitiser binding at the gate electrode and the semiconducting channel. The threshold increases to more positive values; however, it remains below the gate voltage V_G_ = 300 mV used to record output characteristics, such that all outputs are recorded above threshold and, therefore, within the validity of Equation (1). Specific capacitance is reduced. Consequently, according to Equation (1), both responses lead to a reduction in drain current. Our WGTFT-based RBD spike protein sensor thus combines both capacitive and potentiometric responses into an amplified drain current response. As further merit of delineation, our procedure can in principle reveal if potentiometric and capacitive response would affect I_D_ in different directions, e.g., by shifting threshold into ‘normally on’ behaviour, thus weakening the overall response of I_D_ to analyte. This situation could then be remedied by using a hole-transporting semiconductor instead, reversing the trend between I_D_ and V_T_ shift.

### 4.3. Modelling Response Characteristics

**Figure 6:**
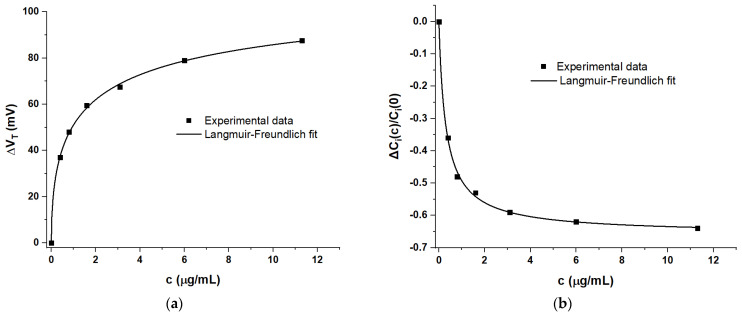
(**a**) Potentiometric response metric ΔV_T_(c) and (**b**) capacitive response metric ΔV_T_(c) and ΔC_i_(c)/C_i_(0) plotted against RBD spike protein concentration, c, in the sample pool.

For a more quantitative analysis of potentiometric and capacitive response, the response metrics, ΔV_T_(c) and ΔC_i_(c)/C_i_(0), are separately plotted against concentration in Figure 6 below, to complement the prior Figure 4.

Both response metrics, as well as the previously introduced drain current metric ΔI_D_(c)/I_D_(0) (Figure 4), were fitted using OriginLab software to a model based on the Langmuir‒Freundlich (LF) isotherm, as shown in Equation (6):

ΔI_D_(c)/I_D_(0) = [ΔI_D_(∞)/I_D_(0)] Θ(c) (6a)

ΔV_T_(c) = ΔV_T_(∞) Θ(c) (6b)

ΔC_i_(c)/C_i_(0) = [ΔC_i_(∞)/C_i_(0)] Θ(c) (6c)

Θ(c) = (kc)^β^/[(kc)^β^ + 1] (6d)

Within the LF model, Θ(c) describes the fraction of available surface adsorption sites occupied by sorbate (here, the analyte) when an adsorbing surface (here, the sensitiser) is in contact with a liquid that carries sorbate at concentration, c. k is the association constant of the sorbate/adsorption site interaction and is related to enthalpy of adsorption, ΔH, and temperature, via a Boltzmann factor. The parameter β describes inhomogeneity in k for different sites. In the absence of inhomogeneity, β = 1 retrieves the classic Langmuir surface adsorption isotherm. The Langmuir and LF models have been employed previously to describe the potentiometric response of some WGTFT sensors based on either the adsorption of small organic molecules on a sensitiser surface [8], or ion-exchanging sensitisers [12,13,17]. The LF model is mathematically equivalent to the equation introduced by Hill in 1910 [28], which has ever since been routinely used for the quantitative description of biochemical interactions (e.g., [29]). Note that the Hill equation is formulated with the dissociation (rather than association) constant of the sorbate/adsorption site interaction, k_D_ = 1/k, which gives it a different appearance to Equation (6). As the biochemistry literature uses somewhat confusing nomenclature (Hill vs. Hill‒Langmuir equation), here, we use the equivalent, but unambiguously named LF model. The parameters resulting from fitting response metrics to Equation (6) are summarised in Table 2.

It is evident from Figure 4 and Figure 6 that the LF model provides a good fit to all amperometric, potentiometric, and capacitive sensor metrics. The good match of data to the LF model allows us to extrapolate the maximum possible change of capacitance: ΔC_i_(∞)/C_i_(0) = −0.659; i.e., under very high spike protein concentration, the EDL capacitance drops to about one-third of its initial value. This is similar in order-of-magnitude to previous reports, e.g., [16]. However, it is evident from Table 2 that the fit parameters k and β do not agree between potentiometric and capacitive sensor metrics. The amperometric metric convolutes threshold and capacitance shift and is affected by the somewhat arbitrary choice to read I_D_ at V_G_ = V_D_ = 0.3 V and, therefore, may differ in its parameters. However, the discrepancy between potentiometric and capacitive LF parameters is at variance with the conventional interpretation of Θ(c) as fractional occupation of sorbent (sensitiser) sites. This conclusion is accessible only because of the simultaneous presence, and successful delineation, of potentiometric and capacitive responses.

The observed discrepancy in LF parameters warrants further analysis. We note that, like almost all proteins in biofluids [3], the RBD spike protein is electrically charged: according to Pawlowski [29], RBD spike carries an overall electric charge of +2 elementary charges at physiological pH = 7.4. Despite its much larger size than ‘simple’ ions (e.g., metal cations), it is tempting to model the potentiometric response to spike protein against the Nikolsky‒Eisenman (NE) law widely used to describe potentiometric ion sensors, Equation (7) (e.g., [14]):

ΔV_T_(c) = 59/z mV log(a/a_st_ + 1) = s log(a/a_st_ + 1) (7)

where z is the valency of the ion, a is its activity, and a_st_ sets a limit-of-detection (LoD). Activities usually are of similar magnitude to ion concentration, c, and, by definition, approach them exactly in the limit c → 0. As activity coefficients for RBD spike protein are not yet established, we approximate a, a_st_ by c, c_st_; i.e., we assume unit activity coefficient. Equation (7) is derived from the classic Nernst law, but avoids its unrealistic divergence in the limit c → 0. For c >> c_st_, Equation (7) implies a linear response with log c with a slope of s = 59 mV/z per decade in c. For c << c_st_, ΔV_T_(c) flatlines towards ΔV_T_(c) → 0 for c → 0. At c ≈ c_st_, the threshold shifts versus log c curves upwards from a flat line (c << c_st_) towards a line of slope s = 59 mV/z (c >> c_st_). At exactly c = c_st_, ΔV_T_(c_st_) = s log 2 ≈ 18 mV/z.

**Figure 7:**
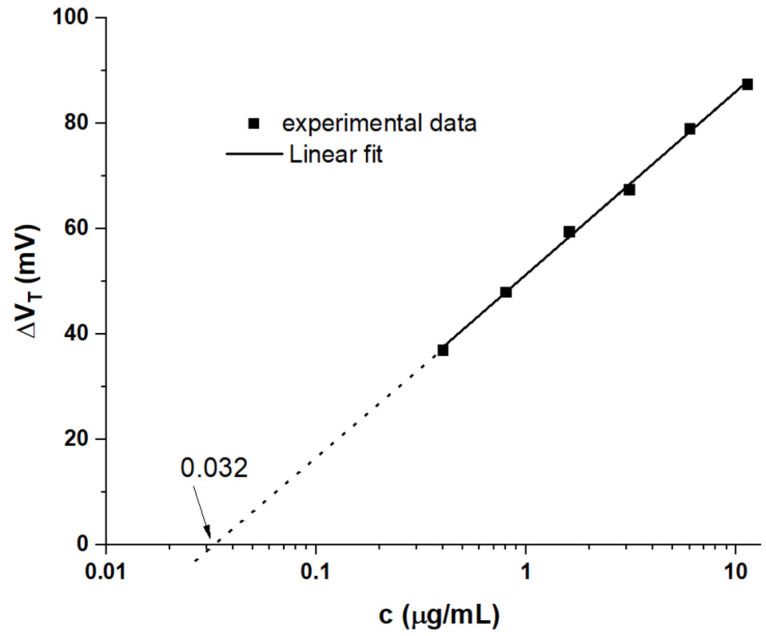
Potentiometric response metric ΔV_T_(c) against a logarithmic RBD spike concentration scale. The intercept corresponds to c_st_.

The range of concentrations studied here covers ≈ 1.5 decades, which is somewhat narrow on a logarithmic scale. However, within that limited range, a logarithmic potentiometric response law as suggested by Equation (7) provides a good fit, as shown in Figure 7.

Note that a corresponding plot for ΔC_i_(c)/C_i_(0) (not shown here) is not fitted well by a straight line, which is unsurprising as Equation (7) only applies to potentiometric sensors.

The fitted line in Figure 7 displays a slope of s = (34.8 ± 0.7) mV/decade, suggesting an NE law with an (average) z of 1.7 elementary charges per spike protein. This is close to the z = 2 reported by Pawlowski [29]. The small discrepancy may be due to the non-unity activity coefficient. Further, we note that the thin film transistor threshold follows Equation (8) [27]:

V_T_ = V_FB_ + V_SAM_ − (D_f_ + D_t_)/C_i_ (8)

where V_FB_ is the flat band voltage; V_SAM_ is the potential of a self-assembled monolayer (here, the sensitiser immobilised on the gate contact); D_f_ and D_t_ are the electric displacement (charge/unit area) due to fixed (f) and trapped (t) interface charges, respectively, at the semiconductor surface; and C_i_ again is the specific capacitance of the gate medium. The NE law only describes V_SAM_, but it is evident from Equation (8) that C_i_ also affects V_T_. While C_i_ is constant for sensors responding to ‘small’ inorganic ions, here, we have shown that C_i_ declines with increasing RBD spike protein concentration, which, according to Equation (8), indirectly affects V_T_ as well. This may lead to an overall shift of V_T_ somewhat in excess of the NE law, which is formally accounted for by z < 2.

Extrapolation of the fitted line in Figure 7 to the intercept with ΔV_T_(c) = 0 gives an estimate of c_st_ as 32 ng/mL. This is somewhat larger than the c_LoD_ = 6.2 ng/mL evaluated from the amperometric metric, cf. Figure S3. The amperometric sensor metric is amplified by combining both potentiometric and capacitive responses, while c_st_ is evaluated from potentiometric response alone, hence the former can reach a lower limit-of-detection. We note that, for c << c_st_, overall sensor response would indeed be capacitive only—not because of screening in the electrolyte, but because of the flatlining of the NE law. However, this only holds for c << c_st_ and, hence, small changes of capacitance; that is, at c_st_, we calculate ΔC_i_(c_st_)/C_i_(0) = −0.0685 with Equation (6c) and the parameters from Table 2.

## 5. Conclusions

We report on an SnO_2_-based WGTFT gated across a pool of a high ionic strength buffer solution as electrolyte with an Au gate contact sensitised by the prior immobilisation of the antibody (immunoglobuline) against the SARS-CoV-2 virus. As analyte, we titrate aliquots of the RBD spike protein of the SARS-CoV-2 virus into the buffer pool. We find that an increasing RBD spike concentration in the gate pool strongly impacts the WGTFT output and transfer characteristics, making our WGTFT a viable sensor for RBD spike protein. As the immunoglobuline binds to the virus via the viruses’ spike protein, this observation in all likelihood translates to the SARS-CoV-2 virus itself. However, here, we avoid working with the full virus as it represents a major biohazard. A WGTFT for the SARS-CoV-2 virus close to clinical applications has already been reported by Seo et al. [10], but without the delineation between potentiometric and capacitive response, which is the focus of our study. 

When we use a simple response metric based on WGTFT drain current, we can quantify RBD spike protein with a limit-of-detection of 6.2 ng/mL. To distinguish between the underlying potentiometric and capacitive response mechanisms, we introduce an analysis based on output characteristics. Outputs are recorded at constant gate voltage, bypassing issues resulting from gate voltage dependency of transistor parameters, as discussed in detail in the introduction. We find a parallel potentiometric and capacitive response, both acting to reduce drain current and thus amplifying WGTFT drain current response over a purely capacitive—or a purely potentiometric—sensor.

Parallel potentiometric and capacitive response contrasts with prior reports on antibody-sensitised WGTFT biosensors claiming a purely capacitive response owing to screening of the potentiometric response in ‘strong’ (high ionic strength, i_s_) electrolytes when the analyte/sensitiser binding site is distanced from the transistor’s semiconducting channel by more than the electrolyte’s Debye length [7,9,16]. The authors followed the reasoning of Reed et al. [30] that, owing to the large size of the sensitiser, the analyte/sensitiser binding site is distanced from the semiconducting channel by more than the Debye screening length in typical biological fluids. For example, in phosphate buffered saline (PBS), which is a common simulant for physiological electrolytes like blood, ionic strength i_s_ = 163 mmol/L and Debye length 0.76 nm [9]. Screening is said to limit the WGTFT biosensor response to a purely capacitive mechanism; that is, the large sensitiser introduces an additional serial capacitance into the gate medium that is modulated by analyte/sensitiser binding. Charts of capacitive sensor metric versus analyte concentration c are reported in [7,9,16], but not fitted to a response model.

In contrast, here, we clearly observe potentiometric response in a high i_s_ electrolyte with sensitiser immobilised at the gate electrode, which distances it from the semiconducting channel by far more than the Debye length, as is always the case in gate-sensitised WGTFTs. The longest possible Debye length in water is 971 nm, due to the ionic strength resulting from autoprotolysis, 2H_2_O ⇄ H_3_O^+^ + OH^−^, while WGTFT gates are separated from the channel by distances in the order mm. Our findings here agree with the work of Melzer et al. [31], who also report a potentiometric response in a WGTFT sensitised at the gate contact, with no evidence of screening. Our observations also agree with the work of Guo et al. [32], who reported a WGTFT biosensor for the H5N1 avian flu virus with an unusual design; that is, the antibody to H5N1 virus is immobilised as a sensitiser on the top surface of an (inorganic) semiconductor, but gating is from below, across a strong electrolyte soaked into a microporous SiO_2_ film. This design makes the sensor ‘blind’ to capacitance changes at the top semiconductor/sensitiser surface. Nevertheless, Guo et al. report a strong potentiometric response in excess of 300 mV under H5N1 avian flu virus.

We find the screening reasoning inconsistent: WGTFTs always communicate the gate potential to the semiconducting channel across an electrolyte over distances far beyond the Debye length, unaffected by the claimed screening. We have no doubt of the presence of a screening effect in the work of Reed et al. [30], but note a key difference between the WGTFT and the design used by Reed et al. That is, in their work, the electrolyte is not electrically contacted at all, rather it is electrically ‘floating’ without a defined potential. In the WGTFT design, the electrolyte is addressed by the gate electrode, which defines a potential at the point of contact. The screening reasoning cannot be transferred from the floating electrolyte in [30] to the contacted electrolyte in the WGTFT.

Instead, we believe the prior conclusions are partly based on artifacts resulting from the analysis of transfer rather than output characteristics, which relies on the unrealistic assumption of gate voltage independent carrier mobility and specific capacitance, as discussed in the introduction.

Parallel potentiometric and capacitive response mode enhances sensitivity and reduces the limit-of-detection. Empirically, both capacitive and potentiometric response metrics found here can be modelled well by a LF response law, which is mathematically equivalent to the Hill equation that is often fitted to biosensor response characteristics, e.g., [26]. However, LF parameters do not match between potentiometric and capacitive responses. We find that the potentiometric response is also modelled well by the Nikolsky‒Eisenman (NE) law, Equation (7), treating the RBD spike protein as an ion. This is in spite of its much larger size and weight (234 amino acids, molecular weight ≈ 30 kDa [23]) than inorganic ions, e.g., metal cations, for which the NE law was developed. There are, however, limits to the generality of this observation. On the one hand, the NE law fails for non-ionic analytes (z = 0 in Equation (7)). Nevertheless, WGTFTs with potentiometric response to some non-ionic organic molecules have been reported [8] and fitted to a LF law. On the other hand, a full virus rather than a single spike protein carries many elementary charges, z >> 1, which, according to Equation (7), should lead to a negligible potentiometric response. Still, Guo et al. [32] find a strong potentiometric response linear with c in a WGTFT sensitised for the H5N1 avian flu virus.

As overall recommendations for future work with transistor-based biosensors, we recommend the WGTFT (electrolyte is contacted) over the ISFET (electrolyte is electrically floating) design to enable parallel potentiometric and capacitive responses; we recommend to avoid organic semiconductors in the WGTFT channel because of their instability and gate voltage-dependent carrier mobility; and we recommend to record and analyse output rather than transfer characteristics, using the procedure introduced here.

## Figures and Tables

**Table 1:** Results of quantitative evaluation of the output characteristics, Figure 2: axis intercepts V_DX_ and (I_D_/V_D_)_X_ from the I_D_/V_D_ vs. V_D_ plot, resulting in V_T_ and ^W^/_L_ μC_i_(c) calculated with Equation (3) and Equation (4), and sensor metrics ΔV_T_ and ΔC_i_(c)/C_i_(0), as defined in Equation (5), against RBD spike concentration, c.

c (μg/mL)	V_DX_ (mV)	(I_D_/V_D_)_X_ (mA/V)	V_T_	^W^/_L_ μC_i_(c) (mA/V^2^)	ΔV_T_	ΔC_i_(c)/C_i_(0)
			(mV)		(mV)	
0	550	1.6	25	5.8	0	0
0.4	476	0.88	62	3.7	37	−0.36
0.8	454	0.69	73	3	48	−0.48
1.6	431	0.57	84.5	2.7	59.5	−0.53
3.1	415	0.49	92.5	2.4	67.5	−0.59
6	392	0.43	104	2.2	79	−0.62
11.3	375	0.4	112.5	2.1	87.5	−0.64

**Table 2:** Parameters from the fitting of the LF model, Equation (6), to the response metrics shown in Figure 4 and Figure 6.

	Parameter	ΔI_D_(∞)/I_D_(0)	ΔV_T_(∞)	ΔC_i_(∞)/C_i_(0)	k	Β
Metric			(mV)		(mL/μg)	
Amperometric		−0.83 ± 0.01	-----	-----	3.97 ± 0.15	0.85 ± 0.05
Potentiometric		-----	120 ± 11	-----	0.57 ± 0.21	0.53 ± 0.06
Capacitive		-----	-----	−0.659 ± 0.018	3.16 ± 0.25	0.94 ± 0.13

## Data Availability

Not applicable.

## Supplementary Materials

The following are available online at https://www.mdpi.com/article/10.3390/s21165618/s1, Section S1: WGTFT characteristics at different pool fill level, Section S2: Transfer characteristics on logI_D_ scale, Section S3: LoD for RBD spike from LF fit, Section S4: Estimating carrier mobility in SnO_2_.
