# Supplementary material for: Parallel Potentiometric and Capacitive Response in a Water-Gate Thin Film Transistor Biosensor at High Ionic Strength
Source: Sensors (Basel). 2021 Aug 20;21(16):5618. doi: 10.3390/s21165618 (PMC8402598; doi:10.3390/s21165618)
Supplement: Supplementary file 1 [file sensors-21-05618-s001.zip › sensors-1318298-supplementary.pdf]

sensors

MDPI

S1: WGTFT characteristics at different pool fill level

As a control experiment, we recorded output characteristics before and after pipetting an additional 40 µL of buffer into the gate pool already filled with 500 µL Certipur buffer, slightly raising the fill level. This mimics the later addition of analyte to the gate pool when the WGTFT is addressed by a sensitised gate contact.

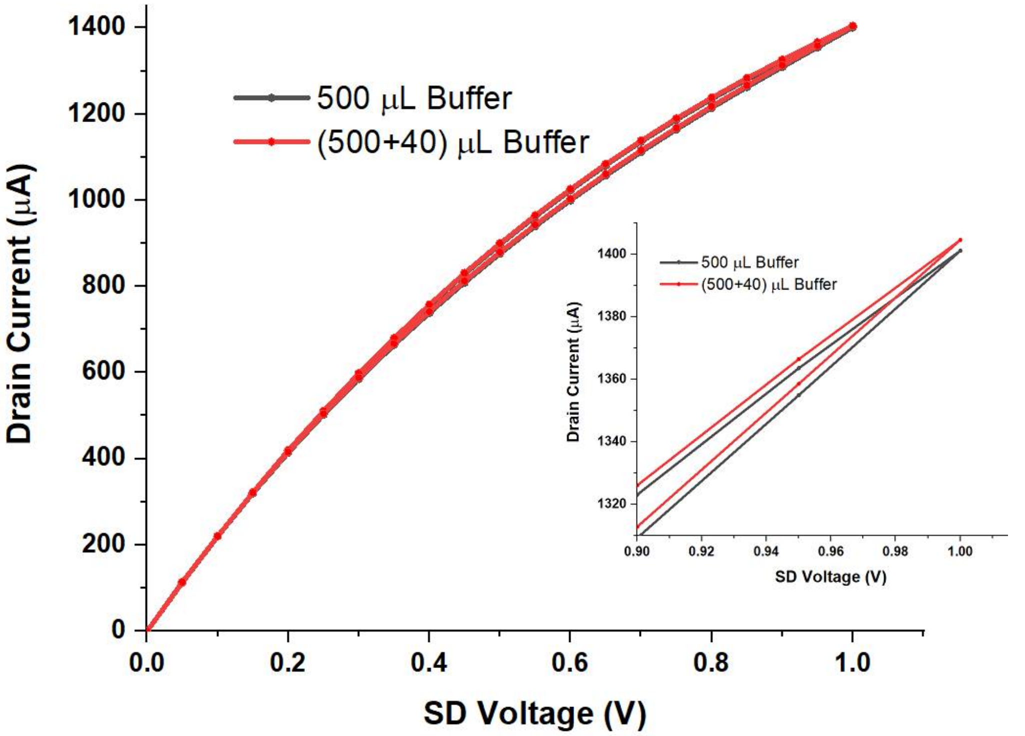

Figure S1. Outputs at V_G = 0.3 V for a WGTFT gated with 500 µL and (500 + 40) µL = 540 µL buffer in the gate pool. The difference between the characteristics is minimal, such that they appear superimposed on each other. Only a magnification (zoom-in, inset) reveals a small difference.

Figure S1 shows that the addition of some more buffer to the gate pool has almost no impact on the recorded output characteristics (I_D changes by ~1% or less). Later observations of the impact of adding analyte to the gate pool can thus safely be assigned to the binding of analyte to sensitised gate contact, rather than merely to a raising of the fill level in the pool.

S2: Transfer characteristics on logI_D scale

The transfer characteristics shown in Figure 3 are re-plotted on a logarithmic scale below.

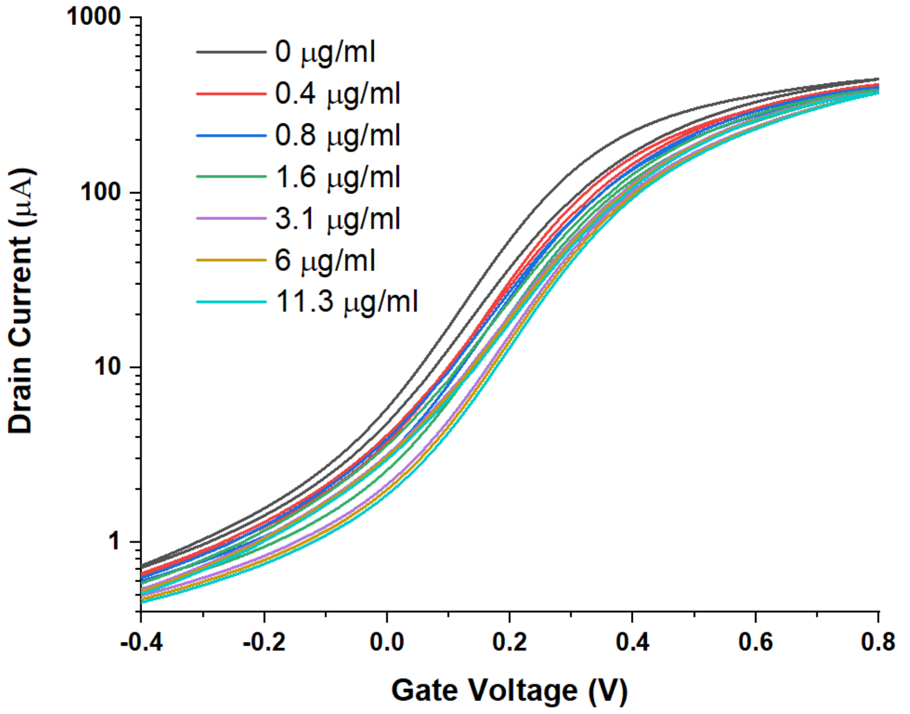

Figure S2. The transfer characteristics from Figure 3 plotted on logI_D scale.

A quantitative evaluation of WGTFT subthreshold behaviour is difficult because there may be leakage currents in the aqueous medium. These are negligible compared with the 'true' (transistor channel) source → drain currents above the threshold, but become comparable in magnitude in the subthreshold regime, and are emphasised by the logarithmic scale. We do, however, conclude from Figure S2 that the WGTFT is above the threshold at $V_G = 0.3$ V for all analyte concentrations; that is, the logI_D versus V_G characteristics are clearly curved at $V_G = 0.3$ V. Below the threshold, logI_D versus V_G would follow a straight line. Moreover, transfers at different analyte concentrations are near-parallel to each other at small gate voltages. This means that subthreshold swing is not a useful sensor metric here.

S3: LoD for RBD spike from LF fit

To estimate a limit-of-detection with the help of an LF fit, we follow the procedure described in [20], Figure 5. We transform the LF response, Equation (6a), by multiplying with the denominator of Θ(c), Equation (6d), and plot $[\Delta I_D(c)/I_D(0)]\cdot[(kc)^{\beta} + 1]$ versus $(kc)^{\beta}$, using the parameters k and β from Table 2. This linearises the response law; the plot is shown below.

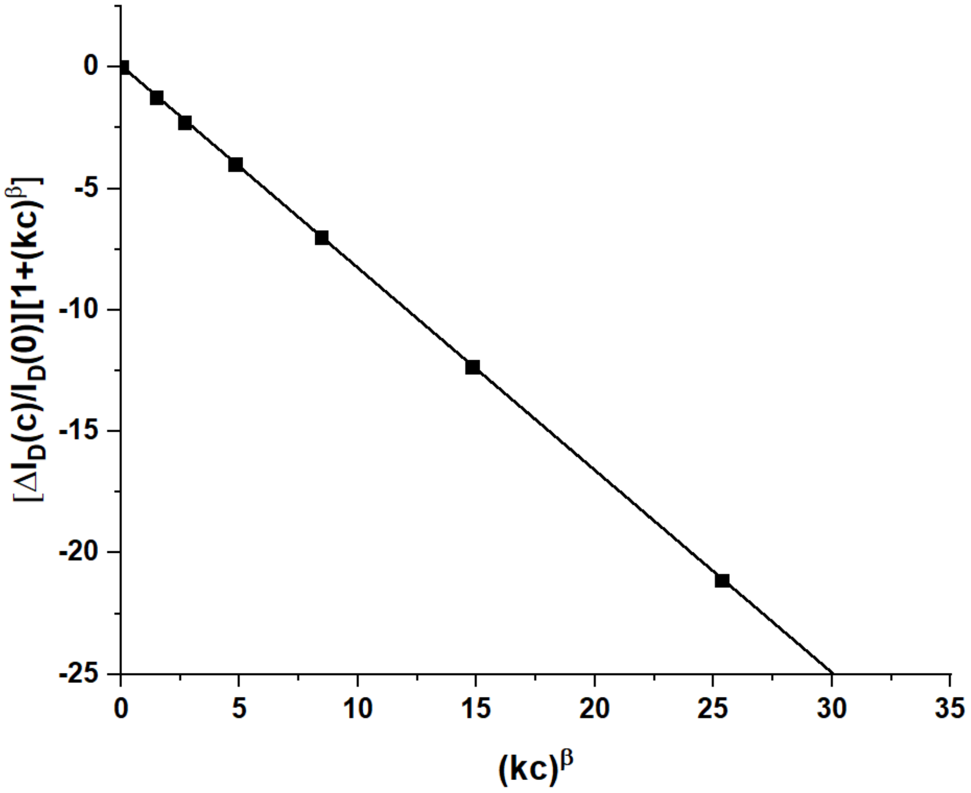

Figure S3. Linearised plot of drain current response to RBD spike vs. concentration, $[\Delta I_D(c)/I_D(0)]\cdot[(kc)^\beta + 1]$ vs. $(kc)^\beta$.

We fit a straight line to the transformed data and find slope m = –0.833, and intercept b +/- Δb = 0.00652 +/- 0.012. As expected, Δb > b because the LF law predicts a zero intercept. Δb is an estimated standard error that allows calculating LoD with the common '3 standard errors' criterion:

$$(kc_{LoD})^\beta = 3\Delta b/|m| = 0.04322 \quad \text{S1)}$$

With knowledge of k, β from Table 2 and Δb, m from Figure S3, c_{LoD} can be evaluated from Equation S1 as c_{LoD} = 6.2 ng/mL.

S4: Estimating carrier mobility in SnO_2

For the spray-pyrolysed SnO_2 WGTFT under buffer, but in the absence of analyte (c = 0), Table 1 shows $^W/_L\ \mu C_i(c = 0)$ = 5.8 mA/V². Here, $^W/_L$ = 33.3 ⇒ $\mu C_i = 1.756 \times 10^{-4}$ A/V². μC_i is about five times larger than implied by Figure 2a in [8] for another SnO_2 WGTFT, but note that the different gating setup and much lower ionic strength in [8] probably lead to lower C_i.

To extract mobility from the mobility–capacitance product, we need an estimate for the capacitance of EDLs in buffer solution. Zhao et al. [26] treat the EDL as a capacitor with the dielectric constant of water, and the thickness equal to the Debye screening length, to estimate $C_i \approx 3$ μF/cm². Note this relies on the Guoy–Chapman EDL model and does not account for a Helmholtz layer. However, several authors agree on $C_i \approx 3$ μF/cm² for aqueous EDLs at low frequency, somewhat independent of ionic strength, including DI water [5], where the Guoy–Chapman model strongly underestimates DI water EDL capacitance.

Assuming C_i = 3 μF/cm² = 0.03 F/m², we calculate

$$\mu C_i = 1.756 \times 10^{-4}\ \text{A/V}^2 \quad \Rightarrow \quad \mu = (1.756 \times 10^{-4}/0.03)\ \text{m}^2/\text{Vs} = 59\ \text{cm}^2/\text{Vs}$$

Note that both the mobility evaluated here and μC_i in Figure 2a in [8] relate to the linear regime, albeit here we evaluated an output characteristics, while Figure 2a in [8] is a linear transfer characteristic. The mobility estimated here is thus consistent, or somewhat better, but in the same order, as reported previously in the literature.
